# Body Composition and Spasticity in Children with Unilateral Cerebral Palsy—A Case–Control Study

**DOI:** 10.3390/children9121904

**Published:** 2022-12-05

**Authors:** Lawia Szkoda, Andrzej Szopa, Andrzej Siwiec, Ilona Kwiecień-Czerwieniec, Małgorzata Domagalska-Szopa

**Affiliations:** 1 Department of Physiotherapy, Medical University of Silesia in Katowice, 40-752 Katowice, Poland; 2 Neuromed, Rehabilitation and Medical Center in Katowice, 40-698 Katowice, Poland; 3 John Paul II Pediatric Center in Sosnowiec, 41-218 Sosnowiec, Poland; 4 Department of Developmental Age Physiotherapy, Medical University of Silesia in Katowice, 40-751 Katowice, Poland

**Keywords:** spastic hemiplegia, segmental body composition, spasticity of lower limbs

## Abstract

The aim of this study was to identify the correlations between segmental body composition and the spasticity level of the affected lower limb in children with unilateral cerebral palsy (spastic hemiplegia). Additionally, an attempt was made to identify the differences in composition between the affected and unaffected lower limbs using segmental body composition analysis. This case–control study included 31 children with spastic hemiplegia aged 8 to 16 years with differing severities of spasticity in the lower limbs. The reference group consisted of a control group which included 31 peers with corresponding age and sex to the tested group. Negative correlations obtained in the statistical analysis showed that higher spasticity level in the iliopsoas muscle is associated with lower limb fat-free mass and lower limb muscle mass. Our results showed that children with spastic hemiplegia have worse parameters of body composition in the affected limb than in the unaffected one. To confirm the importance of these results, further studies are needed in a larger population which includes non-ambulatory children.

## 1. Introduction

Symptoms of cerebral palsy are a primary or secondary consequence of central nervous system damage in the early stage of development. Primary symptoms mostly manifest as incorrect development of posture and movement such as gross motor skills (gross motor function), small motor skills (eye–hand coordination and hand function), and articulation. In contrast, secondary disorders are related to incorrect muscle tone development and include muscle spasticity, contractures of muscles and soft periarticular tissues (ligaments and joint capsules), and bone deformations (subluxations and dislocations of limb joints, chest deformations, and scoliosis). Individuals with postural and motor control disorders that are not the main clinical symptoms—according to definition—are not considered to have cerebral palsy [1]. On the basis of the localization/limb distribution of neuromotor impairment in spastic CP, three basic forms of cerebral palsy can be distinguished: quadriplegia (severe spasticity of all four limbs), diplegia (involvement of the four limbs but greater spasticity and weakness in the lower limbs), and hemiplegia (involvement of the upper and lower limbs on one side of the body [1].

Although symptoms of unilateral paresis vary across individuals, they mostly are manifestations of upper and lower limb function disorders on the same side of the body and may be right sided or left sided depending on the location of the central nervous system damage. Limb paresis occurs on the side opposite to the side of damage (in case of damage to the right hemisphere of the brain, paralysis occurs in the left limbs and vice versa). In most patients, the upper limbs are more involved than the lower limbs [1]. A serious manifestation in children with unilateral cerebral palsy is spasticity, which is caused by damage to the cortical movement control centers that impairs conductivity between the nervous and muscular system and causes muscle tone balance dysfunction between antagonistic muscle groups [2]. The characteristic feature of motor control disorders is intensified manifestations of spasticity in the distal rather than proximal limb segments; therefore, distal muscles are more vulnerable to the effects of spasticity. Consequently, tonic activity in paretic muscles increases, and the growth of these muscles during development becomes limited. It causes contractures of muscles and soft periarticular tissues (ligaments and joint capsules), leading to limitation in range of movement and impairment in arbitrary movements of particular joints, mostly of limbs [2]. In addition, cerebral palsy in children is characterized by disordered muscle tone distribution, that is, increased muscle tone (including spasticity) in lower and upper limbs and decreased muscle tone in the head–body axis (central hypotonia), limiting activity of the musculoskeletal system [3,4], which may disturb bone and muscle mass development in children with cerebral palsy. Previous research has shown that both body mass index (BMI) percentile and body composition in children with cerebral palsy are different from those in their healthy peers [3,4,5,6,7,8,9,10,11,12,13,14,15,16,17]. Results of these studies have also shown that, with a higher level of disability defined by Gross Motor Function Classification System (GMFCS) level [4,5,8,9,10], or with the most severe form of cerebral palsy [6,7,11], indices based on BMI are lower.

According to our knowledge, only two studies have shown a correlation between level of spasticity in limb muscles in children with cerebral palsy and their body composition [3,4]. The first one included 118 children with cerebral palsy presenting with different levels of functional ability, representing all GMFCS levels, and with different spasticity levels of muscles of lower limbs, as defined by degrees on the Ashworth scale [4]. The second one was our previous research comparing 59 ambulatory patients with bilateral cerebral palsy (spastic diplegia, GMFCS I and II) to healthy peers [3]. Although the results of these studies revealed correlations between general muscle mass deficiency and spasticity level, the composition of involved lower limbs was not analyzed, and studied dependencies have referred to the correlation between general body composition and the level of spasticity of lower limbs. The encouraging results of these studies led us to perform this research to determine the correlations between lower limb composition and their level of spasticity in children with unilateral cerebral palsy. The basic purpose of this research was to identify the correlations between segmental body composition and the spasticity level of the affected lower limb in children with unilateral cerebral palsy (spastic hemiplegia). Additionally, an attempt was made to identify the differences in composition between the affected and unaffected lower limbs using segmental body composition analysis.

## 2. Materials and Methods

This research is a part of a broader research program on assessment of body composition in children with cerebral palsy, performed to assess quantitative differences in particular body composition components, including lower limbs, and the spasticity level in these patients. The study was approved by the Bioethics Committee of our institution (KNW/0022/KB1/38/18). A group of 31 participants was tested, including children with unilateral cerebral palsy (spastic hemiplegia) aged 8 to 16 years with differing severities of spasticity in the lower limbs. The inclusion criteria were as follows: (1) diagnosed and clinically confirmed unilateral cerebral palsy (spastic hemiplegia); (2) an ability to maintain a standing position; and (3) informed consent of parents or caretakers for participation in the research. The exclusion criteria were (1) presence of other severe and chronic diseases; (2) feeding disorders and swallowing problems (dysphagia); and (3) inability of the child to communicate and follow instructions.

The control group included 31 peers of corresponding age and sex to the tested group from the primary school in Sosnowiec (Poland) and who had no central movement disorders based on medical records. The criterion for inclusion in the control group was a normal body weight with a BMI between 25 and 75 percentile.

The study consisted of three parts: (1) anthropometrical measurements and BMI percentile assessment; (2) total and segmental body composition examination; and (3) assessment of spasticity level in the lower limbs.

Before body composition examination in any participant, height was measured using a TANITA height rod with an accuracy of 0.5 cm. Based on the measurements, the following indices were calculated:

1. BMI: a weight/height index calculated as weight divided by height squared (kg/m^2^);
2. BMI z-score and BMI percentile: calculated using the AnthroPlus application for age and sex, respectively, according to the World Health Organization (WHO) reference.

Then, using the TANITA scale MC-780 S MA (TANITA Corporation, 1-14-2 Maeno-cho, Itabashi-ku, Tokyo 174-8630 Japan), total and segmental body composition (TBC and SBC, respectively) examinations were performed [18].

During the examination, the patient was in underwear, standing straight on the scale in a relaxed position. All measurements were performed around midday after a light breakfast eaten around 9.00 a.m. and before lunch, which was usually eaten between 11.00 and 13.00. The following parameters of TBC were analyzed [3,19]: basal metabolic rate (kJ); fat mass (kg); percentage of fat mass (%); fat-free mass (FFM, kg); percentage of fat-free mass (FFM%); fat-free mass index (kg/m^2^); total body water (TBW, kg); percentage of total body water (TBW%); muscle mass (kg); percentage of muscle mass (%); skeletal muscle mass (kg); percentage of skeletal muscle mass (%); impedance (IMP, Ohm); and sarcopenic index (kg/m^2^). The following SBC parameters for the left and right lower limbs [19] were assessed: lower limb fat percentage (LFM%), lower limb fat mass (LFM, kg), lower limb fat-free mass (LFFM, kg), lower limb fat-free percentage (LFFM%), lower limb muscle mass (LPMM, kg), lower limb muscle percentage (LPMM%), and lower limb impedance (LIMP, Ohm). In further analysis, the following body mass rates were assessed: BMI calculated using the TANITA scale, percentile BMI and z-score BMI calculated by the AnthroPlus program (according to the recommendation of the WHO) [20], and BMI OLAF (reference distributions of height, weight, and BMI for Polish children and adolescents) centile charts developed by the Institute “Pomnik” Child Health Center [21]. Additionally, participants were categorized as 1—underweight (<25 c), 2—normal (25–75 c), or 3—overweight and obese (>75 c) according to the WHO Growth References [22,23].

Next, the spasticity level of the lower limbs in the tested group was analyzed using Ashworth’s six-degree scale with Bohannon and Smith modification (Modified Ashworth Scale; MAS) [24]. Each MAS assessment was performed once by the same experienced physiotherapist who was blinded to both total and segmental body composition examination results. The following muscle groups were scored on a scale from 0 to 4: (1) iliopsoas, (2) anterior thigh muscles, (3) medial thigh muscles, (4) posterior thigh muscles, and (5) posterior shin muscles, where 0 was scored for a normal muscle tone, 1 for a slight increase in muscle tone perceptible as a resistance at the end of flexion and extension, +1 for a slight increase in muscle tone with a resistance for less than a half of the range of movement, 2 for a significant increase in muscle tone perceptible as resistance during the full range of movement, 3 for a significant increase in muscle tone, making passive motion significantly limited, and 4 for contracture in flexion or extension (Table 1) [24]. The average degree of spasticity intensity was calculated for the right and left lower limbs and both lower limbs separately.

### Statistical Analysis

Differences in mass composition between the affected and unaffected lower limbs were determined in the tested group. For this purpose, the right and left lower limbs were considered as the affected and unaffected limbs, depending on hemiplegic side. To compare body composition between the tested and control groups and between the affected and unaffected lower limbs, paired Student’s *t*-test was used. To compare nonparametric variables, sex, and BMI classification, the chi-square test was used. Data are presented in tables as mean, standard deviation, minimum, and maximum. In all analyses, statistical significance was approved at α = 0.05.

To assess the influence of limb type on (affected/unaffected/healthy) particular lower limb composition, one-way analysis of variance was performed with Dunn–Bonferroni post hoc analysis.

Further, nonparametric rho Spearman correlations were performed because variables related to limb spasticity are expressed on an ordinal scale. Only correlation rates with α = 0.05 were considered statistically significant. Correlation rates were interpreted in accordance with Altman’s recommendations: <0.2, poor; 0.21–0.4, low; 0.41–0.6, moderate; 0.61–0.8, high; and 0.81–1, very high. Statistical analysis was performed using RStudio 1.2.1 program (250 Northern Ave, Boston, MA, USA).

## 3. Results

Demographic and anthropometric data of the tested and control groups are shown in Table 1. Mean height, weight, BMI, BMI z-score, BMI percentile, OLAF BMI, OLAF height, and OLAF weight were statistically lower in children with unilateral cerebral palsy (tested group) than in children of the control group.

Because no participant was overweight or obese (BMI percentile >75 c), both the tested and control groups were divided into two subgroups: (1) thin and (2) normal (Table 2). Most children with unilateral cerebral palsy were underweight (71% of the tested group), while normal values were evident only in nine children (29%). The control group did not have any underweight children. All participants in the control group presented with a normal BMI (100%).

Comparative analysis of significant differences in TBC parameters between the tested and control groups is shown in Table 3. Children with cerebral palsy exhibited significantly lower values for almost all general body composition parameters, except higher FFM% and TBW%, and insignificant differences in IMP.

Although, overall, the comparative analysis of the SBC of the lower limbs in children with cerebral palsy did not show significant differences in mass composition between the affected and unaffected lower limbs, LPMM% was statistically lower in the affected lower limbs than in the unaffected lower limbs (Table 4). The comparative analysis of the SBC of the lower limbs in children of the control group did not show significant differences between the right and left lower limbs.

For better identification of differences in SBC between the affected and unaffected lower limbs, SBC comparison was performed between the affected and unaffected lower limbs in children with cerebral palsy (n = 31) and healthy limbs in children of the control group (n = 62) (Table 5). Results of the Dunn–Bonferroni post hoc analysis showed significant differences between the healthy limbs and the affected and unaffected limbs of analyzed participants. LFFM (kg), LPMM (kg) and LPMM%, and LIMP (Ohm) in the healthy lower limbs were statistically higher than in the affected and unaffected limbs. These results confirmed that LPMM (%) in the unaffected lower limbs in the tested group was statistically higher than in the affected lower limbs.

The analysis of spasticity severity in lower limb muscles showed that the highest spasticity severity was observed in the posterior shin and iliopsoas muscles (Table 6). On the other hand, the lowest spasticity severity was observed in the medial thigh muscles (Table 6). The average spasticity level for the affected lower limb was almost 2 points (Table 6).

Next, the correlation between spasticity in the affected lower limbs in children with unilateral cerebral palsy and particular components of mass composition in these limbs was assessed. Results of the correlation analysis showed no significant correlation between the average value of spasticity and the particular parameters of mass composition of the affected lower limbs (Table 7).

Lastly, the correlation between the mass composition of the affected lower limbs and spasticity of particular muscle groups in the affected lower limbs was determined (Table 8). Results of the correlation analysis showed moderate, negative correlations of LFFM (kg) and LPMM (kg) with spasticity of the iliopsoas muscle (Table 8). The negative values of the correlation show that the higher spasticity level in the iliopsoas muscle was associated with lower FFM and muscle mass in the affected limb.

## 4. Discussion

The main goal of this research was to assess the correlations between the level of spasticity of muscles in the affected lower limb and body composition in children with unilateral cerebral palsy. To achieve this goal, we defined the TBC and SBC of affected and unaffected lower limbs, as well as the spasticity level of particular muscle groups of the affected lower limb.

The results of the TBC examination obtained in this study confirmed the differences in general body mass composition between children with cerebral palsy and their healthy peers identified in previous studies [3,4,5,6,7,8,9,10,11]. We found that the majority of children with unilateral cerebral palsy were underweight (71% of the tested group). Children with unilateral cerebral palsy also exhibited lower values of almost all general body mass composition parameters than children in the control group. Although previous research did not specifically include children with unilateral cerebral palsy, our results correspond with the results of previous research carried out in children with cerebral palsy. In our previous research [3], comparative analysis between ambulatory children with CP and their healthy peers showed that children with CP presented with significantly lower values of the majority of body composition components such as basal metabolic ratio and percentage of fat mass, skeletal muscle, and muscle mass [3,4]. Finbraten et al. reported that children with GMFCS level III and IV showed growth inhibition and had higher fat mass and lower FFM than ambulatory children (GMFCS I and II) [5]. Sung et al. also confirmed that children with cerebral palsy had significantly lower FFM, soft lean mass, skeletal muscle mass, and bone mineral content than their healthy peers. Additionally, some authors showed that non-ambulatory children (GMFCS IV to V) had worse results for the above-mentioned parameters than ambulatory children with cerebral palsy (GMFCS I to III) [10].

The results of the SBC of the affected lower limbs in children with hemiplegia obtained in this study were interesting. The comparative analysis of the affected and unaffected lower limbs in children with unilateral cerebral palsy generally did not show differences in particular components of lower limb mass, but muscle mass in the affected limbs (LPMM%) was significantly lower than in the unaffected limbs. Additionally, the LFM (kg), LFFM (kg), and LPMM (kg) of the affected limbs in children with unilateral cerebral palsy were significantly lower than that of the lower limbs in their healthy peers. Similar results were obtained in our previous study conducted in children with diplegia [3]. Affected limbs were characterized by significantly lower LFFM and LPMM than the limbs of healthy peers [3]. Asymmetrical body posture in children with hemiplegia means that the affected lower limb is usually not fully loaded, which may lead to muscle mass deficiencies in the affected lower limb compared with the unaffected limb.

To the best of our knowledge, these are the only publications on this subject in the literature, which makes it impossible to refer to the results of other authors. Indirectly, we may refer to the results obtained by Macedo et al., who showed significant differences between the affected and unaffected halves of the body in the case of thigh and midcalf skin folds in the group of adults with hemiplegia [25].

The most important finding of this study was the correlation between body composition and spasticity level in particular muscle groups in the affected limbs. Negative correlations obtained in the analysis showed that a higher spasticity level in the iliopsoas muscle is associated with lower LFFM and LPMM. It is worth mentioning that spasticity level in the iliopsoas muscle in the affected lower limb was, on an average, 2.15 on the five-point Ashworth scale and was lower only than the spasticity in the posterior lower leg muscle group (2.81). Because the main function of this muscle is flexion in the hip joint, its spasticity limits extension in the hip joint, plays a significant role in the asymmetrical pelvis position, and contributes to inadequate weight bearing on the affected limb [26]. The lack of symmetrical weight bearing on both lower limbs may be fundamental for diagnosis in this study, as differences in mass composition between the affected and unaffected lower limbs and correlations between the spasticity of iliopsoas muscle and LFFM and LPMM in the affected lower limb were observed. Although the observed correlations cannot show a cause–effect relationship, we may infer that spasticity of the iliopsoas muscle may play a primary role in the physical activity of children with unilateral spastic cerebral palsy.

Our results partially confirm those of Więch et al., who showed that children with cerebral palsy and a higher severity of spasticity (on the Ashworth scale) had lower fat mass, FFM, muscle mass, and TBW [4]. Because, according to our best knowledge, our study is the only study that attempted to determine the correlations between body mass composition and spasticity level in particular groups of muscles in the affected lower limb, it is not possible to compare our findings with those of other studies in the literature.

To conclude, though a few previous studies have showed worse general body composition parameters in children with cerebral palsy than in their healthy peers, the results of our study showed that children with unilateral cerebral palsy have lower values of parameters of body composition in the affected limb than in the unaffected one. Additionally, we found reverse correlations between the level of spasticity in the iliopsoas muscle and FFM and muscle mass in the affected lower limb. However, despite the promising results of this study, further research is needed in a larger population of children with cerebral palsy.

We acknowledge the limitations of this study. Even though we analyzed the body composition of children with unilateral cerebral palsy in a wide age range, that is, from 8 to 16 years, the stage of puberty and level of physical activity of participants were not specifically evaluated, which may have influenced the weight, body composition, and potential differences between the study groups. The frequency of occurrence of unilateral cerebral palsy in children and the rigoristic inclusion criteria did not allow the inclusion of a larger study population. Another limitation was the inclusion of only those children who were able to maintain the standing position due to the approved method of body composition measurement used. It led to the exclusion of several children with unilateral cerebral palsy. Therefore, within the limitations of this study, the following conclusions could be drawn:

## 5. Conclusions

1. Children with unilateral cerebral palsy exhibit greater deficits in general muscle mass than their healthy peers;
2. Children with unilateral cerebral palsy are characterized by lower muscle mass in the affected limbs than in the unaffected limbs, as well as by lower muscle mass in affected lower limbs in comparison with the lower limbs of healthy peers;
3. The obtained results showed no significant correlation between the mean value of spasticity and particular parameters of mass composition of the affected lower limbs;
4. To define the correlations of the level of spasticity in the affected lower limb muscles with skeletal muscle mass in children with unilateral cerebral palsy, further studies are needed in a larger population which includes non-ambulatory children.

## Figures and Tables

**Table 1:** Modified Ashworth Scale procedure.

Muscle	Patient Position	Testing Procedure
Iliopsoas	Supine, lower limb in flexion	Affected lower limb moved through the range of available hip extension
Anterior thigh muscles	Prone, lower limb in extension	Distal limb moved through the range of available knee flexion
Medial thigh muscles	Supine, lower limb in midline	Extended lower limb moved through the range of available hip adduction
Posterior thigh muscles	Supine, lower limb distal to the knee was suspended over the plinth edge	Distal limb moved through the range of available knee extension
Posterior shin muscles	Supine, leg in midline	Foot moved through the range of available ankle dorsiflexors

**Table 2:** Demographic and anthropometric data of the tested and control groups.

Parameter	Tested Group (N = 31)		Control Group (N = 31)		Statistical Test
					*p*-Values
Age (years); M ± SD, range	11.74 ± 2.73	8–16	11.74 ± 2.73	8–16	*t* = 0.00; 1.00
Height (cm); M ± SD, range	145.02 ± 13.71	119.50–170.00	155.50 ± 14.51	128.00–182.50	*t* = −2.92; 0.00
Weight (kg); M ± SD, range	36.53 ± 10.51	22.40–60.00	54.20 ± 9.58	40.00–75.70	*t* = −6.92; 0.00
BMI; M ± SD, range	17.08 ± 2.67	12.45–24.10	22.34 ± 1.42	18.60–24.40	*t* = −9.70; 0.00
BMI z-score; M ± SD, range	−0.72 ± 1.19	−3.58–0.86	0.53 ± 1.08	−1.46–2.65	*t* = −4.32; 0.00
BMI percentile; M ± SD, range	39.78 ± 34.12	0.10–97.90	62.61 ± 27.90	7.20–99.60	*t* = −2.88; 0.01
OLAF BMI; M ± SD, range	21.13 ± 33.13	0.10–97.00	59.29 ± 24.85	7.00–89.00	*t* = −5.13; 0.00
OLAF height; M ± SD, range	25.03 ± 32.48	0.10–99.90	53.51 ± 32.17	1.00–99.90	*t* = −3.47; 0.00
OLAF weight; M ± SD, range	29.11 ± 29.22	0.10–96.00	59.19 ± 27.43	4.00–97.00	*t* = −4.18; 0.00
Girls; N (%)	21 (68)		20 (65)		χ^2^ = 0.07; 0.79
Boys; N (%)	10 (32)		11 (35)		
GMFCS level I; N (%)	5 (16.00)				
GMFCS level II; N (%)	26 (84.00)		−		
BMI classification:			−		
1; N (%)	22 (71.00)		0 (0.00)		
2; N (%)	9 (29.00)		31 (100.00)		

M, mean; SD, standard deviation; range, minimum–maximum; *t*, Student’s *t*-test; *p*, *p*-value; χ^2^, chi-square test; BMI, body mass index; GMFCS, Gross Motor Function Classification System; OLAF, reference distributions of height, weight, and BMI for Polish children and adolescents; BMI classification: (1) thin (<10 c); (2) normal (25–75 c).

**Table 3:** Descriptive characteristics of total body composition parameters and statistical analysis.

Parameter	Tested Group (N = 31)		Control Group (N = 31)		*t*	*p*
	M ± SD	Min–Max	M ± SD	Min–Max		
BMR (kJ)	4769.64 ± 481.19	4006.80–5400.20	6045.78 ± 551.17	5534.08–7900.00	−9.88	0.00
FM (kg)	7.51 ± 4.36	1.30–18.60	12.81 ± 3.70	7.30–19.00	−5.16	0.00
FM%	19.31 ± 7.08	3.00–28.10	23.02 ± 5.36	12.10–32.30	−2.33	0.02
FFM (kg)	29.32 ± 8.64	15.00–45.00	36.85 ± 6.96	23.30–50.00	−3.78	0.00
FFM%	80.69 ± 7.08	71.90–97.00	76.98 ± 5.36	67.70–87.90	2.33	0.02
FFMI (kg/m^2^)	13.60 ± 2.13	8.88–18.22	15.14 ± 1.19	13.18–17.75	−3.51	0.00
TBW (kg)	21.17 ± 7.32	3.50–37.60	27.45 ± 5.15	20.10–40.00	−3.90	0.00
TBW%	0.59 ± 0.08	0.20–0.70	0.51 ± 0.04	0.43–0.64	4.62	0.00
PMM (kg)	27.92 ± 8.89	15.00–45.30	35.39 ± 7.50	22.10–52.00	−3.58	0.00
PMM%	65.26 ± 7.86	55.22–93.19	75.98 ± 8.18	58.37–92.84	5.26	0.00
SMM (kg)	14.77 ± 4.09	7.10–29.00	21.73 ± 2.96	17.00–25.90	−7.68	0.00
SMM%	40.79 ± 7.71	22.90–59.20	46.32 ± 9.26	24.80–70.20	−2.55	0.00
IMP (Ohm)	723.98 ± 59.47	600.00–797.00	694.35 ± 66.12	606.63–870.58	1.85	0.07
SMI (kg/m^2^)	4.81 ± 0.69	3.60–6.00	5.71 ± 0.46	5.00–6.94	−6.19	0.00
BONEM (kg)	1.57 ± 0.41	1.00–2.80	1.93 ± 0.28	1.40–2.50	−4.12	0.00
Phase angle (°)	4.94 ± 0.26	4.50–5.60	5.48 ± 0.49	4.60–6.84	−5.42	0.00

M, mean; SD, standard deviation; Min, minimum; Max, maximum; *t*, Student’s *t*-test; *p*, *p*-value; BMR, basal metabolic rate; FM, fat mass; FM%, percentage of fat mass; FFM, fat-free mass; FFM%, percentage of fat-free mass; FFMI, fat-free mass index; TBW, total body water; TBW%, percentage of total body water; PMM, muscle mass; PMM%, percentage of muscle mass; SMM, skeletal muscle mass; SMM%, percentage of skeletal muscle mass; IMP, impedance; SMI, sarcopenic index; BONEM, bone mass.

**Table 4:** Descriptive characteristics of segmental body composition of the affected and unaffected lower limbs of children in the tested group with statistical analysis (Student’s *t*-test).

Parameter	Unaffected Lower Limb (N = 31)	Affected Lower Limb (N = 31)	*t*	*p*	95% CI		d Cohena
	M ± SD	M ± SD			LL	UL	
LFM (kg)	1.77 ± 0.83	1.92 ± 0.96	0.66	0.51	−0.31	0.61	1.33
LFM%	30.92 ± 5.18	32.92 ± 5.51	0.89	0.38	−1.51	3.93	1.13
LFFM (kg)	3.96 ± 1.50	3.87 ± 1.50	−0.25	0.81	−0.85	0.67	1.00
LFFM%	69.08 ± 5.18	67.87 ± 5.51	−0.89	0.38	−3.93	1.51	1.13
LPMM (kg)	3.81 ± 1.53	3.55 ± 1.30	−0.72	0.47	−0.98	0.46	1.38
LPMM%	66.99 ± 5.79	62.70 ± 6.06	−2.85	0.01	−7.30	−1.28	1.10
LIMP (Ohm)	331.76 ± 47.11	345.14 ± 40.88	1.19	0.24	−9.03	35.79	1.33

M, mean; SD, standard deviation; *t*, Student’s *t*-test; *p*, *p*-value; 95% CI, confidence interval; LL and UL, lower and upper limits of the confidence interval; d Cohena, the size of the effect; LFM (kg), lower limb fat mass; LFM%, lower limb fat percentage; LFFM (kg), lower limb fat-free mass; LFFM%, lower limb fat-free percentage; LPMM (kg), lower limb muscle mass; LPMM%, lower limb muscle percentage; LIMP (Ohm), lower limb impedance.

**Table 5:** One-way analysis of variance model.

Parameter	Tested Group (N = 31)				Control Group (N = 31)		Mean Square	F	*p*
	Unaffected Lower Limb (N = 31)		Affected Lower Limb (N = 31)		Lower Limbs (N = 62)				
	M ± SD	Min–Max	M ± SD	Min–Max	M ± SD	Min-Max			
LFM (kg)	1.77 ± 0.83	0.60–3.30	1.92 ± 0.96	0.60–3.70	2.54 ± 0.87	1.40–4.10	7.70	9.88	0.00 ^b,c^
LFM%	30.92 ± 5.18	20.20–41.30	32.13 ± 5.51	20.00–41.90	30.32 ± 6.16	20.20–39.60	33.96	1.02	0.36
LFFM (kg)	3.96 ± 1.50	1.50–6.60	3.87 ± 1.50	1.60–7.00	5.80 ± 1.10	3.30–8.00	54.89	31.96	0.00 ^b,c^
LFFM%	69.08 ± 5.18	58.70–79.80	67.87 ± 5.51	58.10–80.00	69.68 ± 6.16	60.40–79.80	33.96	1.02	0.36
LPMM (kg)	3.81 ± 1.53	1.50–7.00	3.55 ± 1.30	1.50–6.00	5.50 ± 0.95	3.10–7.30	51.74	35.45	0.00 ^b,c^
LPMM%	66.99 ± 5.79	47.50–80.46	62.70 ± 6.06	45.00–68.42	66.55 ± 6.61	57.14–78.43	188.00	4.77	0.01 ^a,b^
LIMP (Ohm)	331.76 ± 47.11	224.70–399.10	345.14 ± 40.88	257.10–431.00	296.95 ± 32.30	235.00–365.90	28073.48	18.83	0.00 ^b,c^

M, mean; SD, standard deviation; F, F test statistic; *p*, *p*-value; Post hoc, Dunn–Bonferroni post hoc test: ^a^, differences between affected and unaffected lower limbs; ^b^, differences between unaffected lower limbs and lower limbs in the control group; ^c^, differences between lower limbs in control group and affected lower limbs; LFM (kg), lower limb fat mass; LFM%, lower limb fat percentage; LFFM (kg), lower limb fat-free mass; LFFM%, lower limb fat-free percentage; LPMM (kg), lower limb muscle mass; LPMM%, lower limb muscle percentage; LIMP (Ohm), lower limb impedance.

**Table 6:** Descriptive characteristics of the degree of spasticity of the affected lower limbs of children in the tested group.

Parameter	Affected Lower Limb (n = 31)	
	M ± SD	Min–Max
Iliopsoas	2.15 ± 0.45	1.50–3.00
Anterior thigh muscles	2.05 ± 0.52	1.50–3.00
Medial thigh muscles	1.37 ± 0.74	0.00–3.00
Posterior thigh muscles	1.18 ± 0.33	1.00–2.00
Posterior shin muscles	2.81 ± 0.40	2.00–3.00
All	1.91 ± 0.30	1.50–2.50

M, mean; SD, standard deviation; Min, minimum; Max, maximum.

**Table 7:** Spearman correlations between the mass composition and mean spasticity of the affected lower limbs (n = 31).

Parameter	r	*p*
LFM (kg)	−0.26	0.16
LFM%	−0.18	0.32
LFFM (kg)	−0.33	0.07
LFFM%	0.18	0.32
LPMM (kg)	−0.34	0.06
LPMM%	0.23	0.22
LIMP (Ohm)	0.19	0.32

r, Spearman rank correlation coefficient; *p*, *p*-value; LFM (kg), lower limb fat mass; LFM%, lower limb fat percentage; LFFM (kg), lower limb fat-free mass; LFFM%, lower limb fat-free percentage; LPMM (kg), lower limb muscle mass; LPMM%, lower limb muscle percentage; LIMP (Ohm), lower limb impedance.

**Table 8:** Spearman correlations between the mass composition of the affected lower limbs and the spasticity of individual muscle groups of the affected lower limbs in the tested group (n = 31).

Muscle Group	LFM (kg)		LFM%		LFFM (kg)		LFFM%		LPMM (kg)		LPMM%		LIMP (Ohm)	
	r	*p*	r	*p*	r	*p*	r	*p*	r	*p*	r	*p*	r	*p*
Iliopsoas	−0.34	0.06	−0.11	0.56	−0.36	0.04	0.11	0.56	−0.40	0.02	0.02	0.92	0.35	0.06
Anterior thigh muscles	0.01	0.95	0.13	0.49	−0.07	0.69	−0.13	0.49	−0.10	0.61	−0.16	0.39	−0.16	0.39
Medial thigh muscles	−0.14	0.45	−0.09	0.63	−0.26	0.15	0.09	0.63	0.25	0.17	0.21	0.26	0.05	0.77
Posterior thigh muscles	−0.15	0.44	−0.05	0.81	−0.21	0.26	0.05	0.81	−0.21	0.25	−0.01	0.95	0.18	0.35
Posterior shin muscles	−0.08	0.68	−0.20	0.28	−0.04	0.84	0.20	0.28	−0.05	0.81	0.23	0.21	0.07	0.71

r, Spearman rank correlation coefficient; *p*, *p*-value; LFM (kg), lower limb fat mass; LFM%, lower limb fat percentage; LFFM (kg), lower limb fat-free mass; LFFM%, lower limb fat-free percentage; LPMM (kg), lower limb muscle mass; LPMM%, lower limb muscle percentage; LIMP (Ohm), lower limb impedance.

## Data Availability

The data supporting the results of this study are available from the corresponding author upon reasonable request from any qualified investigator.
